# Boundary EEG Asymmetry Is Associated to Linguistic Competence in Vascular Cognitive Impairments

**DOI:** 10.3389/fnhum.2018.00170

**Published:** 2018-05-09

**Authors:** Takashi Shibata, Toshimitu Musha, Yukio Kosugi, Michiya Kubo, Yukio Horie, Mieko Tanaka, Haruyasu Matsuzaki, Yohei Kobayashi, Satoshi Kuroda

**Affiliations:** ^1^Department of Neurosurgery, Graduate School of Medicine and Pharmaceutical Science, University of Toyama, Toyama, Japan; ^2^Brain Functions Laboratory, Inc., Yokohama, Japan; ^3^Department of Neurosurgery, Stroke Center, Saiseikai Toyama Hospital, Toyama, Japan; ^4^Department of Medical Course, Teikyo Heisei University, Tokyo, Japan

**Keywords:** electroencephalogram, language, neuronal activity topography (NAT), neurorehabilitation, asymmetry index, vascular cognitive impairments

## Abstract

**Background and Purpose:** We recently noted a gradual change in the boundary electroencephalography (EEG) oscillation of 7.8 Hz between theta (θ) and alpha (α) bands in response to increased atherosclerosis levels in the elderly. The aim of this study was to investigate the role of boundary EEG oscillations of θ-α bands on cognitive functions in vascular cognitive impairments (VCI) patients.

**Materials and Methods:** We examined 55 patients with VCI in carotid stenosis, and underwent EEG in a resting state with closed eyes for 5 min. The asymmetry index (AI) along homologous channel pairs (e.g., F7-8) was assessed using neuronal activity topography (NAT). AI referring to 10 frequency components ranging from 4 to 20 Hz and neuropsychological assessments including linguistic competence were analyzed.

**Results:** The main findings was that the language score had a positive association with AI in 7.8 Hz at F7-8 and a negative association with AI in 6.3 Hz at C3-4 and 14.1 Hz at F3-4.

**Conclusion:** EEG asymmetry in a boundary range might have a special role in linguistic competence, suggesting the application of neural oscillation on the cognitive function evaluation and neurorehabilition induced by a frequency-specific transcranial alternating current stimulation.

## Introduction

Electroencephalogram (EEG) signals are rich in information about electrical activity of neurons. In general, to signify the majority of EEG used in clinical practice, EEG oscillations are subdivided into the following bandwidths: delta (δ: 1–3 Hz), theta (θ: 4–7 Hz), alpha (α: 8–12 Hz), beta (β: 13–30 Hz), and gamma (γ: > 30 Hz) waves. Recently, we noted a gradual change in the boundary EEG oscillation of 7.8 Hz between θ and α bands in response to increased carotid atherosclerosis in community-dwelling elderly subjects (Shibata et al., 2017). However, the role of EEG frequency of δ, θ, α, β, and γ bands, including the boundary EEG oscillations between δ and θ (3–4 Hz), θ and α (7–8 Hz), α and β (12–13 Hz), β and γ (29–30 Hz), on cognitive function still remains elusive.

The pathophysiology of vascular cognitive impairments (VCI) in carotid atherosclerosis is unclear and might be associated with diabetes. Previous EEG study in mild cognitive impairment (MCI) patients with diabetes found that resting-state functional connectivity is decreased in the alpha, and beta bands, and lower functional connection at intra-hemispheric distance mainly on the left hemisphere, suggesting a heterogeneous disruption of functional network structure (Zeng et al., 2015). Another study in MCI patients with diabetes found that EEG power and coherence are related to neuropsychological tests, and the theta/alpha power ratios in the frontal and left temporal region were significantly higher in MCI patients than controls (Bian et al., 2014). Although the complex biomarkers, relative power and coherence of EEG could be employed to track cognitive function of MCI subjects with diabetes, they used the following frequency bands: delta (1–4 Hz), theta (4–8 Hz), alpha (8–13 Hz), beta (13–30 Hz), not including the boundary EEG oscillations of θ-α bands (7–8 Hz). Therefore, we investigated the role of the boundary EEG oscillations of θ-α bands in VCI, which might have a treatment potentiality using a frequency-specific transcranial alternating current stimulation (tACS).

EEG asymmetry may be affected by anatomical and functional brain abnormalities, such as brain damage and aging. However, the neurophysiological mechanisms of EEG asymmetry are not adequately understood. A recent neurophysiology study found that EEG asymmetry reflects the severity of cognitive decline in patients with VCI (Sheorajpanday et al., 2009, 2014). On the other hand, another study reported that EEG asymmetry of θ oscillations ranging from 6.4 to 7.8 Hz is crucial for language development in children (Kikuchi et al., 2011), and tACS using θ frequency of 6 Hz over the left prefrontal and parietal cortices, which may artificially induce EEG asymmetry, improved cognitive performance in healthy adults (Polanía et al., 2012). In short, conflicting ideas of EEG asymmetry for cognitive recovery or cognitive decline have been reported. If a frequency-specific enhancement of the good cognitive recovery could be found, then tACS would be applied by the specific-frequency oscillation for clinical applications in neurorehabilitation. Therefore, in the present study, to investigate the role of EEG asymmetry and boundary EEG oscillations of θ-α bands on cognitive functions in VCI patients, the relationship between EEG asymmetry ranging from 4 to 20 Hz and linguistic competence was investigated using a neuronal activity topography (NAT).

## Methods

### Subjects (Shibata et al., 2014, 2017)

The selected 55 VCI patients previously admitted at our hospital included 47 men and 8 women aged 58–87 years [mean ± standard deviation (*SD*), 72.6 ± 7.1 years]. Patients were selected based on the following criteria: (i) evidence of unilateral carotid stenosis of >60% (symptomatic or asymptomatic) confirmed with conventional angiography or computed tomography angiography and a degree of carotid artery stenosis determined using the criteria of the North American Symptomatic Carotid Endarterectomy Trial (North American Symptomatic Carotid Endarterectomy Trial Steering Committee, 1991) and (ii) MCI, considered as a score of < 90 (low average) on the Repeatable Battery for the Assessment of Neuropsychological Status (RBANS) (Randolph et al., 1998; Takaiwa et al., 2009). Of the 55 VCI patients with carotid stenosis, 23 had right-sided lesions, 19 had left-sided lesions, and 13 had bilateral lesions. Exclusion criteria included (i) evidence of a previous major stroke and brain damage revealed through MRI; (ii) rapidly evolving symptoms with any hemiparesis, aphasia, and apraxia; and (iii) evidence of dementia, considered as Mini-Mental State Examination (MMSE) score of <24, (iv) history of cerebral surgery, obvious psychiatric or neurological disorders, or (v) uncontrolled or malignant general complications. All VCI patients had good levels of daily living activities.

An objective measure of cognitive performance was obtained with RBANS. In each test, we recorded total scores and individual scores for the five sections or domains of immediate memory (list learning, story memory), visuospatial/constructional (figure copy, line orientation), language (picture naming, semantic fluency), attention (digit span, coding), and delayed memory (list recall, list recognition, story recall, figure recall). Scores were normalized for age, gender, ethnicity, and education level, with a total score of 100 and an *SD* of 15 for the index group.

Fully informed consent in this study was obtained from all patients. The study design was approved by the ethics committee of Saiseikai Toyama Hospital (Toyama, Japan). All subjects gave written informed consent in accordance with the Declaration of Helsinki. All methods were performed in accordance with the relevant guidelines and regulations.

### EEG data acquisition

EEG recordings (EEG-1200/9100; Nihon Kohden Corporation, Tokyo, Japan) were performed in an awake, resting state with eyes closed for 5 min, with 21 electrodes arranged according to the international 10–20 System (Fp1, Fp2, F3, F4, F7, F8, Fpz, Fz, T3, T4, T5, T6, C3, C4, Cz, P3, P4, Pz, O1, O2, and Oz). The contact impedance between the electrode and the scalp was <50 kΩ. The reference electrode was on the right earlobe, and then the average reference is computed as a mean of all electrodes. The raw EEG data were recorded at 0.08–300 Hz with 1,000 Hz sampling rate, and then the converted EEG data for NAT analysis were sampled at 200 Hz per channel and bandpass filtered to pick up signals in a frequency range of 4–20 Hz, which minimizes the effect of artifacts contaminating the recorded signals. The recorded signal sequence was divided into 0.64 s segments. To make the EEG data as high quality as possible and exclude the artifacts, each EEG finding was independently interpreted by EEG specialist who was blind to other data about the subjects except their age and sex. We carefully avoided particular epochs containing ocular movements, baseline shifts, drowsiness signs, and muscle or cardiac contamination.

### EEG data analysis

**Figure 1:**
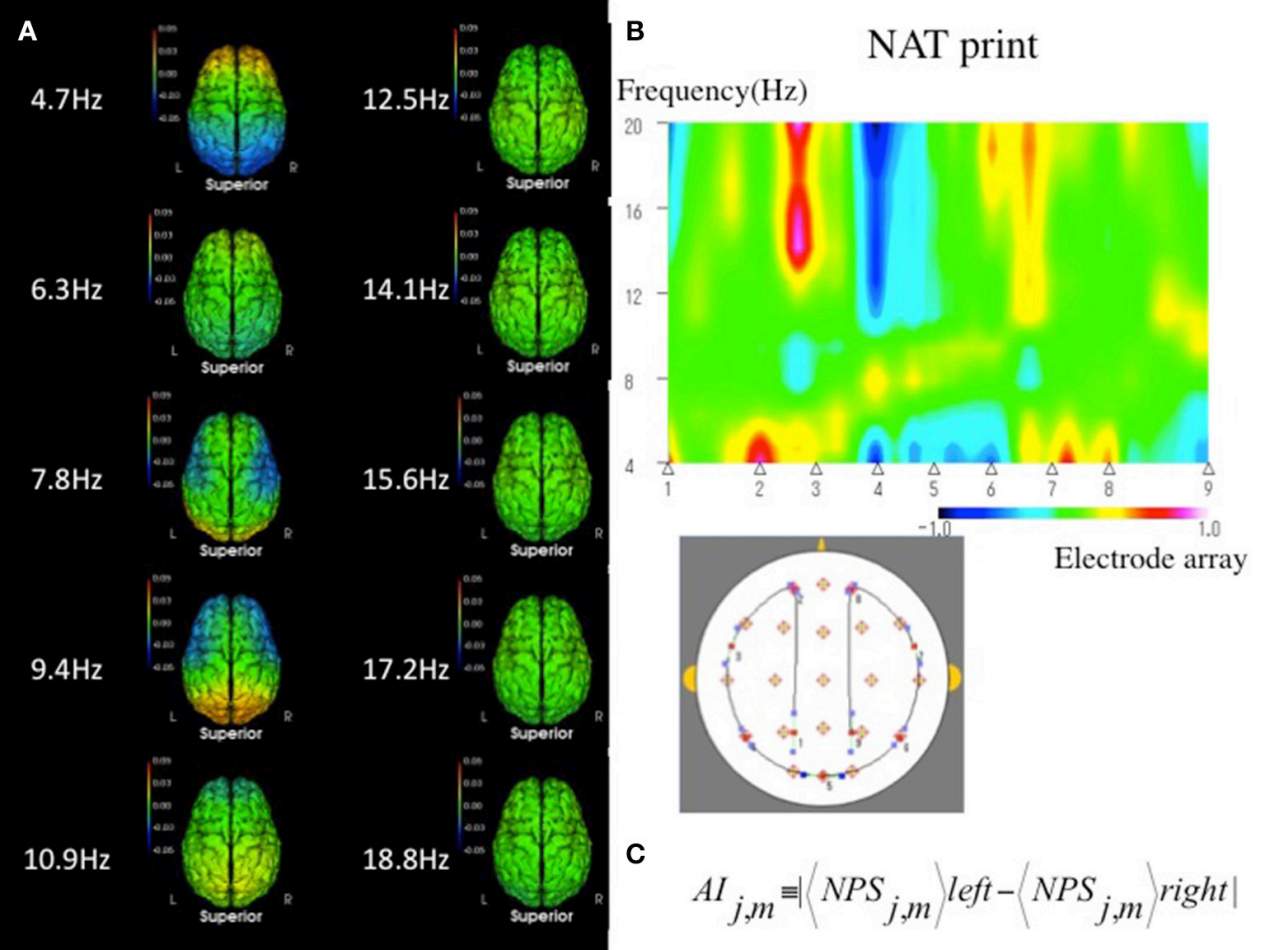
**(A)** Mean sNAT map for a group of 55 VCI patients, viewed from above, for 10 frequency components from 4.7 to 18.8 Hz, which are represented by 210-dimensional NAT spaces. Green indicates average spectrum intensities. Red and blue indicate hyperspectrum intensities and hypospectrum intensities, respectively. L, left; R, right. **(B)** In a qualitative method, NAT print is useful to show geometric symmetrically patterns of EEG on multidimensional NAT spaces at a glance. An example of NAT print symmetrically rearranged at the center of the Oz in a VCI patient. A VCI subject has a tendency to show an asymmetrical pattern in NAT print rather than a normal subject. Electrode array as above 1, 2, 3, 4, 5, 6, 7, 8, and 9 corresponds to P3, Fp1; the middle between F7-T3, T5, Oz, T6; the middle between F8-T4, Fp2, and P4, respectively. Green indicates average spectrum intensity, and red and blue indicate hyperspectrum intensities and hypospectrum intensities, respectively. **(C)** In a quantitative method, interhemispheric asymmetry index (AI) can be measured between the following eight regions and ten frequency components of interest: Eight regions with *j* = 1–8 consists of frontal (Fp1-2, F3-4, F7-8), central (C3-4), temporal (T3-4, T5-6), parietal (P3-4), and occipital (O1-2) pairs, respectively. Ten frequency components with *m* = 3–12 consist of 4.7, 6.3, 7.8, 9.4 10.9, 12.5, 14.1, 15.6, 17.2, and 18.8 Hz, respectively. NPS, Normalized Power Spectrum.

NAT provides rich information about neuronal activities by quantitative EEG analysis (Musha et al., 2002, 2004, 2013; Shibata et al., 2014), which comprises 210 submarkers referring to the 10 frequency components ranging from 4 to 20 Hz. Each submarker has its own role in the characterization of neuronal activities, which are represented with the 210-dimensional NAT spaces. Although NAT may provide quantitative information regarding neuronal activities, it is difficult to interpret all 210 submarkers (Figure 1A). Therefore, we previously used an EEG marker as the likelihood of a test subject to a given template state in VCI (Shibata et al., 2014, 2017), defined as a function of the normalized distance between disease states in the NAT space. However, this likelihood is not applicable enough to assist the physiological assessments because the likelihood includes no local information about neuronal impairments. Therefore, we have introduced another EEG marker of the asymmetry index (AI), which would lead to a more accurate determination of local neuronal impairments than a likelihood score (Figures 1B,C).

The mathematical background of NAT (Brain Functions Laboratory, Inc., Yokohama, Japan) was previously described in detail (Musha et al., 2013).

#### Normalized power spectrum (NPS)

We are going to derive dimensionless markers from the EEG signals which characterize stochastic nature of the neuronal activities generating these signals. The discrete power spectrum (PS) consists of the 10 frequency components ${\langle {\left|X_{j,m}\right|}^{2}\rangle }_{seg}$ for *m* = 3–12 on signal channel *j*. The subscript attached to the averaging symbol denotes, hereafter, that the averaging is performed on this variable. In the present case, the averaging is performed across all of the segments. Dependence of the EEG signal level on individual subject is eliminated by normalizing it to its mean level. The normalized power spectrum consists of ten (*m* = 3–12) such components NPS_*j, m*_ defined as

(1) $$\begin{array}{r} NPS_{j,m}={\langle {\left|X_{j,m}\right|}^{2}\rangle }_{seg}/{\langle {\left|X_{j,m^{\prime }}\right|}^{2}\rangle }_{seg,m^{\prime }} \end{array}$$

We employed m′ as a positive integer running from 3 to 12. Furthermore, in the present study, we employed m in the numerator as the same range of m′ in the denominator. They make a set of ten submarkers for each signal channel. This marker characterizes the fractional partition of the EEG power over the ten frequency components. In short, the NPS is employed as a power ratio between specified frequency and frequency-specific band to reduce the individual differences.

#### Asymmetry index (AI)

Interhemispheric AI-value represents the balance between left and right brain activities which could be easily quantified using NPS. AI was measured between the following eight regions of interest: frontal (Fp1-2, F3-4, F7-8), central (C3-4), temporal (T3-4, T5-6), parietal (P3-4), and occipital (O1-2) pairs. Furthermore, AI was banded into the following ten bands: theta (4.7 Hz, 6.3 Hz, 7.8 Hz), alpha (9.4 Hz, 10.4 Hz, 12.5 Hz), beta (14.1 Hz, 15.6 Hz, 17.2 Hz, 18.8 Hz). Therefore, AI was calculated for each NPS using the following formula:

(2) $$\begin{array}{r} AI_{j,m}\equiv |\langle NPS_{j,m}\rangle left-\langle NPS_{j,m}\rangle right| \end{array}$$

where < *NPS*_*j, m*_ >_*left*_ and < *NPS*_*j, m*_ >_*right*_ were obtained from a left and right channel of a homologous channel pair with j = 1, 2, ···8, which evaluates absolute asymmetry along homologous channel pairs (Fp1-2, F3-4, F7-8, C3-4, T3-4, T5-6, P3-4, O1-2), and at frequency with m = 3,4, ···12, which evaluates the 10 frequency components ranging from 4.68 Hz through 18.72 Hz, respectively.

### Statistical analysis

All data were analyzed by JMP^®^12 (SAS Institute Inc., Japan). Mean ± *SD* values of age, MMSE, RBANS were used as descriptive measures of normally distributed variables. Differences in mean total scores for AI among 3 groups composed of θ bands (4–8 Hz), α bands (8–13 Hz), β bands (13–20 Hz), and five groups composed of frontal lesion (Fp1-2, F3-4, F7-8), central lesion (C3-4), temporal lesion (T3-4, T5-6), parietal lesion (P3-4), occipital lesion (O1-2) were compared by analysis of Tukey's test. Pearson's correlation and multiple linear regression analysis were used for statistical analyses. Firstly, Pearson's correlation was used to determine candidate predictors on the linguistic score. Variables with *P* < 0.05 in Pearson's correlation were included in the multiple linear regression analyses for predicting the language score. Secondly, the stepwise method based on F statistics was used for selection of variables for the final linear regression model. Entry and removal criteria were set at *P* < 0.05 and *P* > 0.10, respectively. Thirdly, ordinary least squares method is used in regression analysis to estimate a best fit model for predicting language scores from AI. Multicollinearity in the model was estimated using a Variance Inflation Factor (VIF), which of greater than five is generally considered evidence of multicollinearity. Differences with a *P* < 0.05 were considered statistically significant.

## Results

### Neuropsychological tests

The mean score (±*SD*) on the MMSE and the RBANS in 55 VCI patients was 27.1 ± 1.5 and 74.5 ± 13.6, respectively. The mean scores of each index including immediate memory, delayed memory, attention, language, and visuospatial/constructional ability were 75.6 ± 15.2, 76.2 ± 16.7, 74.3 ± 14.2, 85.9 ± 6.9, and 87.8 ± 20.3, respectively. Because we were particularly interested in the relationship between EEG asymmetry and linguistic competence, Pearson's correlation was used between the language scores and AI at each channel pair in each frequency band for VCI patients.

### AI

The mean scores (±*SD*) of each AI at frontal (Fp1-2, F3-4, F7-8), central (C3-4), temporal (T3-4, T5-6), parietal (P3-4), and occipital (O1-2) lesions in θ bands (4–8 Hz) were 0.024 ± 0.028, 0.028 ± 0.03, 0.037 ± 0.037, 0.035 ± 0.033, 0.027 ± 0.029, respectively. The mean scores of each AI including frontal (Fp1-2, F3-4, F7-8), central (C3-4), temporal (T3-4, T5-6), parietal (P3-4), and occipital (O1-2) lesions in α bands (8–13 Hz) were 0.015 ± 0.023, 0.02 ± 0.031, 0.022 ± 0.025, 0.024 ± 0.029, 0.016 ± 0.02, respectively. The mean scores of each AI including frontal (Fp1-2, F3-4, F7-8), central (C3-4), temporal (T3-4, T5-6), parietal (P3-4), and occipital (O1-2) lesions in β bands (13–20 Hz) were 0.007 ± 0.008, 0.008 ± 0.009, 0.011 ± 0.01, 0.009 ± 0.009, 0.007 ± 0.007, respectively.

The AI in θ bands (4–8 Hz) was significantly higher compared to that inαbands (8–13 Hz) andβ bands (13–20 Hz), respectively (*P* < 0.0001). The AI in temporal lesions (T3-4, T5-6) was significantly higher compared to that in frontal (Fp1-2, F3-4, F7-8), central (C3-4), and occipital (O1-2) lesions, respectively (*P* < 0.0001).

### Relationship between AI and language score

**Figure 2:**
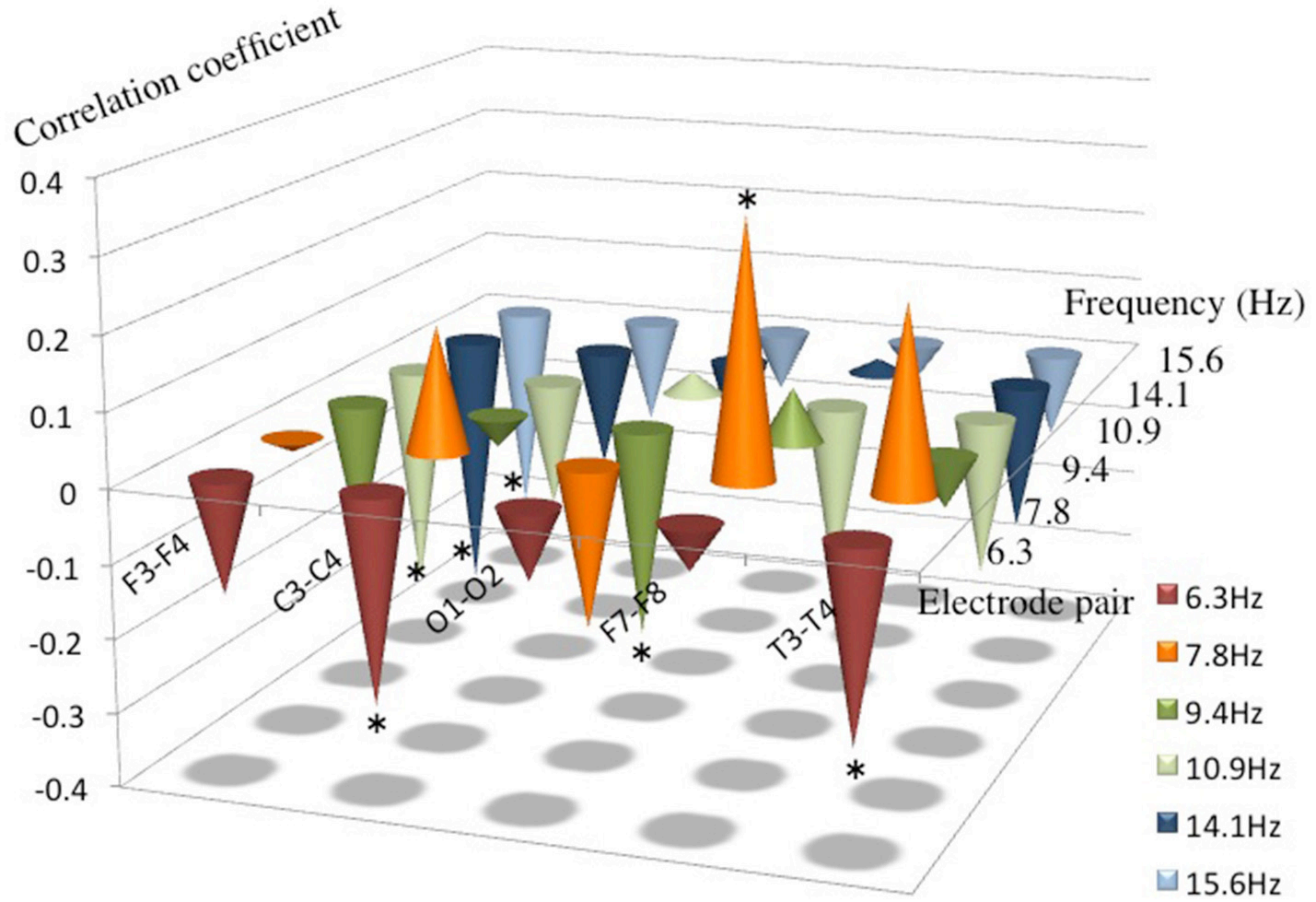
Correlation coefficients between AI from 6.3 to 15.6 Hz and the language score. Note that a positive correlation between AI and the language score was found in 7.8 Hz at F7–8, and a negative correlation was found in 6.3 Hz at T3–4 and C3–4, in 9.4 Hz at O1–2, in 10.9 Hz, 14.1 Hz and in 15.6 Hz at F3–4, respectively. ^*^P < 0.05.

There was a positive correlation of AI in 7.8 Hz at F7-8 and a negative correlation of AI in 10.9 Hz, 14.1 Hz, and 15.6 Hz at F3-4; 6.3 Hz at T3-4; 6.3 Hz at C3-4; and 9.4 Hz at O1-2 channel pair for the language scores (*P* < 0.05), respectively (Figure 2). There were no significant correlations between AI and language scores at each channel pair in each frequency band except the above.

**Figure 3:**
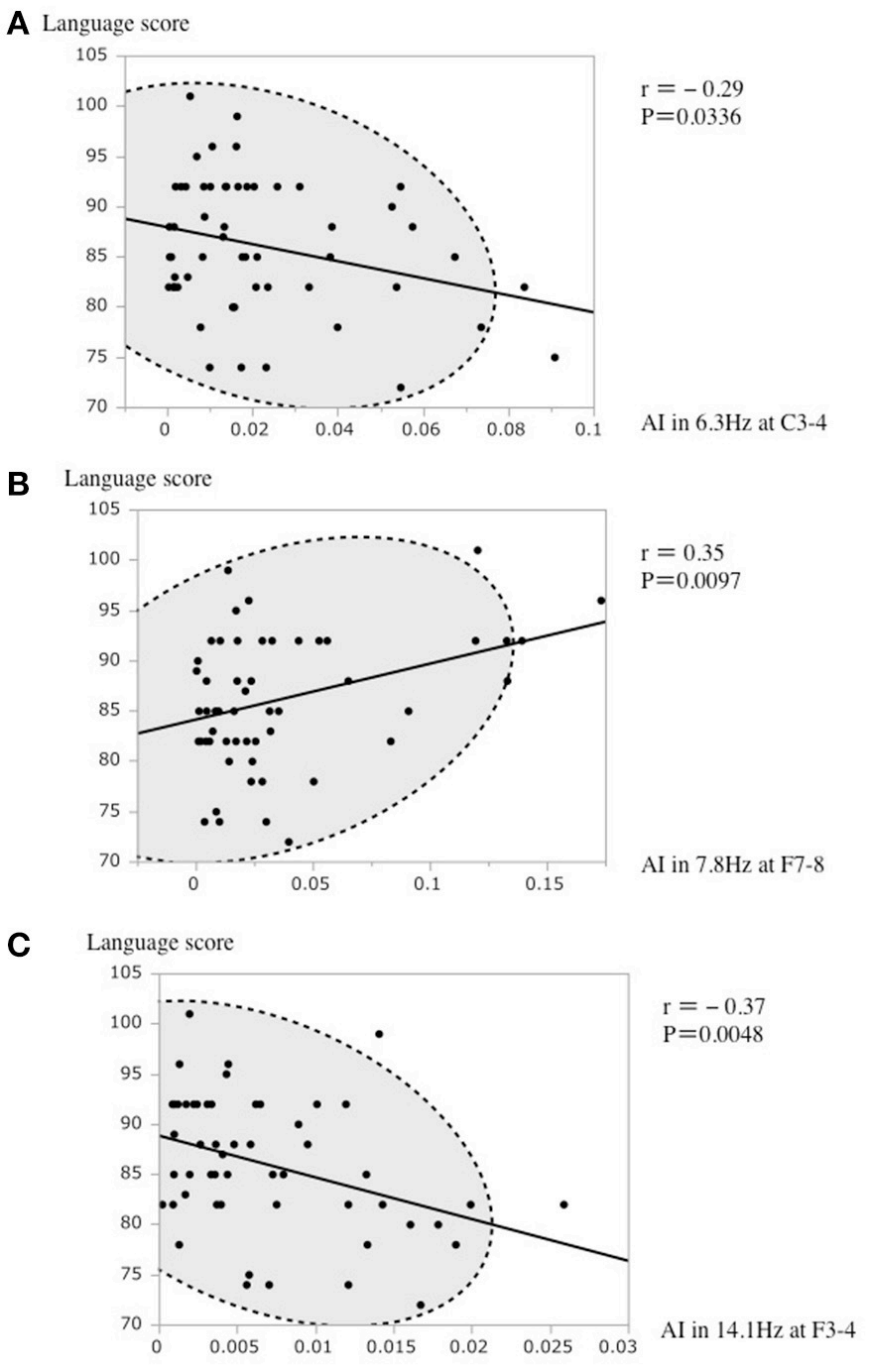
**(A)** A scatterplot of the AI in 6.3 Hz at C3–4 and the language score. **(B)** A scatterplot of the AI in 7.8 Hz at F7–8 and the language score. **(C)** A scatterplot of the AI in 14.1 Hz at F3–4 and the language score. The broken elliptical shape indicates a 95% confidence ellipse for normally distributed data.

In the stepwise regression for selecting the variables for the final linear regression model as above, the language score had a positive association with AI in 7.8 Hz at F7-8 and a negative association with AI in 6.3 Hz at C3-4 and 14.1 Hz at F3-4 (Figure 3).

The regression equation was as follows:

$$\begin{array}{l} LanguageScope\equiv -75\times AI\left(6.3Hz,C3-4\right)+54.2\\ \times AI\left(7.8Hz,F7-8\right)+-387\\ \times AI\left(14.1Hz,F3-4\right)+88 \end{array}$$

(*R*^2^ = 0.32, Adjusted *R*^2^ = 0.28, Root Mean Squared Error = 5.62, *P* < 0.0001).

**Figure 4:**
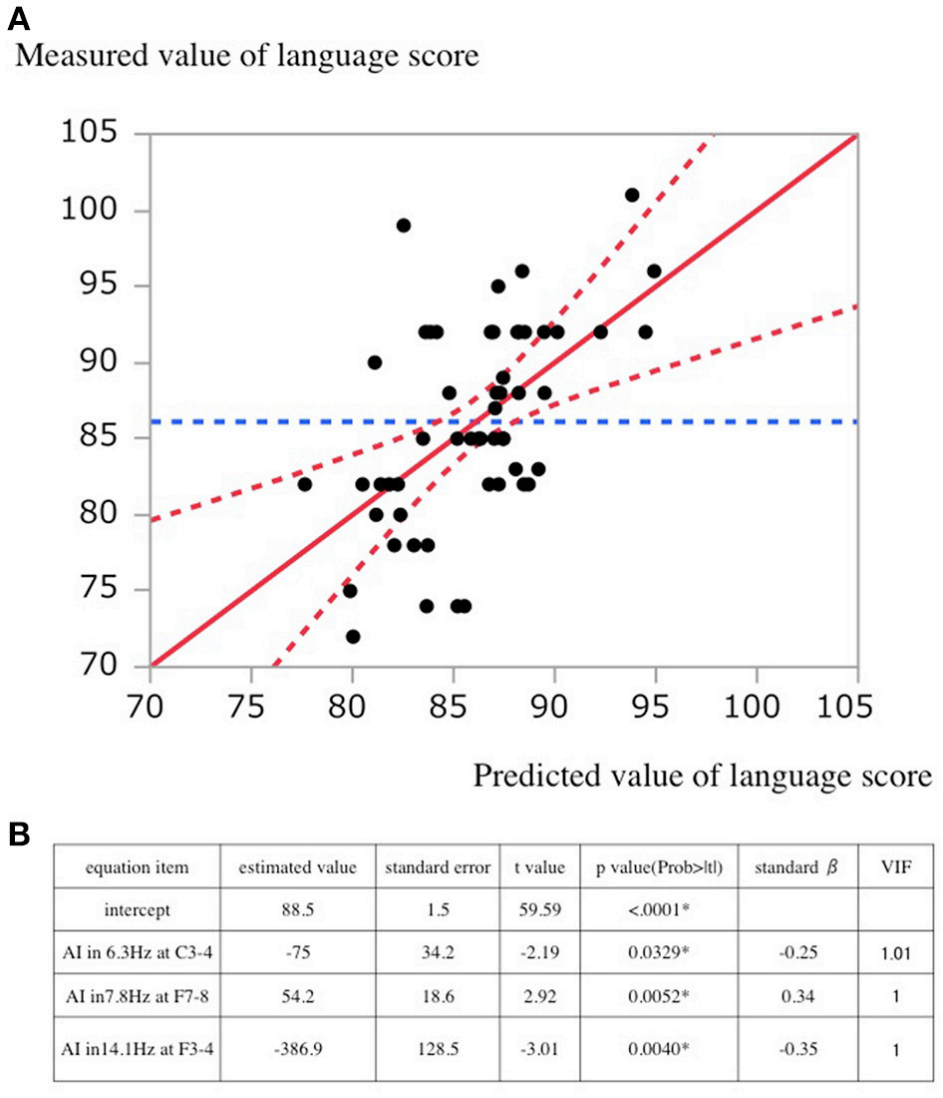
**(A)** A scatterplot of predicted and actual values. The solid lines indicate the regression line. Dashed lines indicate the 95% confidential interval for the regression line. **(B)** Estimated values in the regression equation. standard β, standard partial regression coefficient; VIF, Variance Inflation Factor.

The plot of the actual value and predicted value of language score is shown in Figure 4. The standard partial regression coefficient for AI in 6.3 Hz at C3-4, in 7.8 Hz at F7-8, and in 14.1 Hz at F3-4 was −0.25, 0.34, and −0.35, respectively.

## Discussion

The child's brain has an asymmetrical development of language functions between the two hemispheres, suggesting that a functional asymmetry might reflect positive aspects of language function. Several anatomical and imaging studies have examined whether structural asymmetries underlie this functional lateralization; they have demonstrated a plausible association between functional asymmetries of the brain and language development in children. Functional magnetic resonance imaging (fMRI) studies have demonstrated that dominance language function of the left hemisphere may already be present in infants (Dehaene-Lambertz et al., 2002; Peña et al., 2003). In older children (aged >5 years), and correlations between functional lateralization and language competence have been reported (Balsamo et al., 2002; Ahmad et al., 2003; Everts et al., 2009). In terms of language development, the present study, which has presented a positive correlation between the boundary EEG oscillation of 7.8 Hz and linguistic competence, suggests that the boundary EEG oscillation might be a leftover EEG from childhood with lateralized θ wave connectivity (6.4–7.8 Hz) (Kikuchi et al., 2011).

In general, EEG frequency ranges are divided into θ (4–7 Hz) and α (8–12 Hz) frequency ranges. However, the role of the boundary oscillation (7–8 Hz) between θ and α ranges still remains elusive and has not been adequately investigated. A previous study suggested that the boundary frequency of 6–8 Hz ranges is an anchor range that contributes the development of language ability in young children (Kikuchi et al., 2011). Brain networks in functional lateralization via a boundary EEG oscillation may be crucial for language development. Therefore, we hypothesize that the upper θ oscillation (7–8 Hz), called a boundary (undifferentiated) oscillation, plays an important role in the interplay between θ and α oscillation, differentiates into mature θ and α oscillation with age, and subsequently affects the maintenance of language performance from child to adult. In terms of pathophysiology development, autism spectrum disorder (ASD) may be related with dysfunctional boundary oscillation that does not differentiate into mature θ oscillation ranging from 6 to 7 Hz in preschool children (aged 3–7 years) (Kikuchi et al., 2015).

There are many possible limitations in our study. Firstly, the casual relation between EEG asymmetry and the linguistic competence cannot be determined from our results, because EEG activity is measured at rest when the eyes are closed, which may not necessarily reflect the degree of linguistic competence. Therefore, we cannot predict the degree of language impairment for individual patients using EEG at rest with high precision. Secondly, the neurophysiological mechanisms of EEG asymmetry in aging included psychosocial and pathological factors, which are a more complex process than previously thought. Several studies reported that EEG asymmetry at mid-frontal and mid-lateral frontal regions (F3-4 and F7-8) is a promising neurophysiological marker of depression risk according to the level of trait anxiety (Gollan et al., 2014). In particular, EEG asymmetry was significantly higher in depressed patients than in healthy subjects. Our results may have been partly affected by the degree of a psychologic factor such as a trait anxiety personality type in patients with VCI. Thirdly, although we suggested an association between AI in a narrow band of 1.56 Hz at 7.8 Hz and linguistic competence, our results may have a risk of type I error inflation in multiple parameter testing. Fourthly, our results were exclusively obtained in a population of VCI patients, not normal subjects, and this might prevent any meaningful conclusion about normal EEG rhythms. Therefore, to propose an EEG conjecture about normal EEG rhythms, further prospective studies for normal subjects are needed to confirm our preliminary results.

EEG asymmetry has a potential of providing useful insights into the cognitive recovery using neuromodulation (e.g., tACS) for several diseases, such as depression, ASD, stroke, and VCI. In clinical use of EEG asymmetry, the NAT system may provide supporting quantitative information about the higher degrees of complexity of parameter choice for tACS than that for transcranial direct current stimulation (tDCS). The present study suggests clinical application of tACS therapy for good recovery of cognitive functions, which could induce artificial EEG asymmetry via a specific oscillation of ~7.8 Hz in patients with cognitive impairments. Therefore, we wish to develop an integrated EEG (iEEG) system including neuromodulation and monitor the pre-post EEG changes. In the future, further improvements of the iEEG system for neurorehabilitation are needed to perform tACS, which induces artificial EEG using the most meaningful bands (e.g., ~7.8 Hz) for cognitive and motor recovery; and support an EEG monitor of physical and cognitive interventions to maintain and regain a healthy brain (Bamidis et al., 2014). iEEG system using a specific oscillation based on a tentative theory might efficiently enhance good recovery in patients with cognitive and motor impairments (see Supplemental Information).

## Ethics statement

The ethical committees in the ethics committee of Saiseikai Toyama Hospital. All participants signed a consent form approved by the ethical committees in the ethics committee of Saiseikai Toyama Hospital.

## Data and NAT availability

The data that support the findings of this study are available from the corresponding author upon reasonable request. NAT is supported by Brain Functions Lab., Inc. (Mieko Tanaka, mtanaka@bfl.co.jp).

## Author contributions

TS, TM, YuK, HM, and SK: study design; TS, MK, YH: data acquisition; TS, MT, HM, and YoK: analysis; TS and SK: interpretation of results. All authors contributed to revise and approve the final version of the manuscript and agree to be accountable for this work.

### Conflict of interest statement

The authors declare that the research was conducted in the absence of any commercial or financial relationships that could be construed as a potential conflict of interest.
