# Supplementary material for: Boundary EEG Asymmetry Is Associated to Linguistic Competence in Vascular Cognitive Impairments
Source: Front Hum Neurosci. 2018 May 9;12:170. doi: 10.3389/fnhum.2018.00170 (PMC5954089; doi:10.3389/fnhum.2018.00170)
Supplement: Supplementary file 1 [file Presentation_1.pdf]

Supplemental information

Supplemental information is beyond the scope of the context of the present study, because a mysterious link between EEG oscillations and mathematical constant of circle ratio π in a perfect round is highly speculative and not complete yet. Therefore, if you would be interested in our tentative ideas to complete an iEEG system using a frequency-specific enhancement of cognitive and motor function (Image 1), then we recommend you read as mentioned below and are waiting for your ideas to find a specific-frequency for the good cognitive and motor recovery rather than our proposed-frequency bands.

In the process of discussion in the present study, we noticed by coincidence that approximately 7.8 Hz in boundary EEG oscillation might be a value close to $\pi/4_{(\times 10)}$ (≒ 7.85). Therefore, we propose a tentative theory using an EEG conjecture about a mysterious link between the EEG oscillation and the circle ratio π in the perfect round, according to the purpose of the cognitive and motor recovery.

The law of nature is often written by a mathematical constant such as the circle ratio π, Napier's constant e, and imaginary number i. Although we propose an EEG conjecture about a mysterious link between the boundary EEG oscillations of δ–θ, θ–α, α–β, and β–γ bands and the circle ratio π, $\pi/4_{(\times 10)}$, 4π, and $3\pi^2$, respectively, the conjecture is arbitrary and ambiguous for readers. We selected a mathematically meaningful value, π, as mentioned below (Borwein et al., 2014; Muñoz et al., 2008).

π is an attractive number in the history of mathematics because it is an infinite, nonrepeating floating-point number, implying that every possible number of the date of one's birth (e.g., 14 March) might exist somewhere in π. Therefore, throughout the

history of mathematics (Jonathan et al., 2013), one of the most enduring challenges has been the calculation of π. In the 17th century, Leibniz showed a formula of the slow convergence to π/4. Since the mid-20th century, all calculations of π/4 have been done with the help of calculators or computers. In 1949, the first computer Electronic Numerical Integrator and Computer reached 2037 digits of π/4 using the Machin formula. In 2002, a Japanese team reached 1,241 billion digits of π/4 using the similar Takano formula using a supercomputer. Every country's team has been competing to become the world's best in computing π/4. Interestingly, slow oscillatory stimulation (0.75 ≒ π/4 Hz) over bilateral frontolateral locations during sleep enhances declarative memory performance (Marshall et al., 2006).

There is also a mysterious link between the boundary EEG oscillations of α–β bands and 4π, which is derived from a surface area of a sphere, $4\pi r^2$ (r, radius). Recent EEG data suggests that cortical oscillations in α frequency band (8 – 12 Hz) are correlated with creative thinking and the ability to produce novel and useful work. Interestingly, individuals who generated more original ideas of higher originality in the alternative uses for measuring divergent creative thinking, exhibited a hemispheric asymmetry with stronger increases at the upper α frequency band (10 – 12 Hz) in the right than in the left hemisphere (Fink et al, 2014; Benedek et al, 2011). Alpha synchronization at the upper α frequency band (10.5 – 12.5 Hz) on the right posterior parietal sites may be more specific to creative ideation. According to our hypothesis, tACS using a boundary α–β band (12 – 13 Hz: 4π ≒ 12.56 Hz) over the right frontal–parietal lobe may boost creativity.

In general, the gamma wave is a pattern of neural oscillation in humans with a frequency between 25 and 100 Hz. There is a mysterious link between

the boundary EEG oscillations of β- γ bands and $3\pi^2$ (≒ 29.58 Hz). In particular, the gamma oscillation of 40 Hz is typical. According to a popular theory, gamma waves may be implicated in creating the unity of conscious perception (the binding problem). Recently, the most mysterious number of $4\pi^2$ (≒ 39.43) in mathematical history was found, because $4\pi^2$ is the product over all primes (Muñoz et al., 2008). In clinical use, a recent study reported that gamma frequency (40 Hz) entrainment attenuates amyloid load and modifies microglia in a mouse model of Alzheimer's disease, suggesting new treatments for Alzheimer's disease using gamma frequency (Iaccarino et al., 2016). Interestingly, tACS in the gamma band of 40 Hz during rapid eye movement sleep influences ongoing brain activity and induces self-reflective awareness in dreams, suggesting that higher order consciousness is indeed related to synchronous oscillations around 40 Hz (Voss et al., 2014). However, how gamma waves contribute to the neurodegenerative pathology and self-awareness in dreams remain unclear. On mere supposition, iEEG at $4\pi^2$ Hz might uncover a mysterious link between a gamma oscillation and self-awareness.

To enhance cognitive and motor recovery by transcranial oscillatory potentials, we are going to formulate a clinical trial plan to substantiate a tentative theory about the circle ratio π (Table 1). Furthermore, to imagine a relationship between EEG frequency range and the circle ratio π in the real world, a conceptual diagram is shown in Image 2. Based on the tentative theory, we might effectively select and determine a specific-frequency for good cognitive and motor recovery in neurorehabilitation. Further clinical studies of iEEG are necessary in order to conclude a causal association between a specific-frequency oscillation and cognitive function.

Table 1

Expected clinical effects of specific frequencies for good cognitive and motor recovery.

EEG range (Hz)	Circle ratio	tACS effect	References
Slow oscillations (<1)	$\pi/4 = 0.78$	Memory consolidation	Marshall et al., 2006
δ(1 - 3)		Unknown	
$\delta-\theta$(3 - 4)	$\pi = 3.14$	Unknown	
θ(4 - 7)	$\pi^2/2 = 4.93$	Fluid intelligence	Neubauer et al., 2017
$\theta-\alpha$(7 - 8)	$\pi/4$ (×10) =7.85	Language	Kikuchi et al., 2015
α(8 - 12)	$\pi^2 = 9.86$*	Spatial attention	van Schouwenburg et al., 2017
$\alpha-\beta$(12 - 13)	$4\pi = 12.56$	Creativity	Benedek et al, 2011<br>Fink et al., 2014
β(13 - 30)	$2\pi^2 = 19.72$	Motor function<br>Short-term memory	Feurra et al., 2011<br>Feurra et al., 2016
$\beta-\gamma$(29 - 30)	$3\pi^2 = 29.58$	Unknown	
γ(30 >)	$4\pi^2 = 39.43$	Self awareness<br>Alzheimer's disease	Voss et al., 2014<br>Iaccarino et al., 2016

*Individual alpha frequency (IAF) is well known to vary across individual (Klimesch et al., 1999). Therefore, to use the alpha frequency band, we need to take IAF into account. We will need to discuss an association between the constant of π^2 and variables of IAF in the context of personalized medicine.

Image 1

Schematic overview of an integrated EEG (iEEG)

a) Clinical application of tACS therapy using zero-lag phase synchronization (Polanía et al., 2012) between two distant cortical areas (e.g., between F3 and P3), which may induce artificial asymmetry of EEG via a frequency-specific entrainment of ongoing EEG oscillations. The red (target) electrode is placed on the left frontal (F3) and left

parietal (P3) regions enhanced by entraining synchronous activity within the left fronto-parietal network, while the blue (reference) electrode is placed on the contralateral shoulder.

b) According to the purpose of the cognitive and motor recovery, tACS might be applied at a specific-frequency in δ (1 – 3 Hz), θ (3 – 7 Hz), α (8 – 12 Hz), β (13 – 29 Hz), and γ bands (> 30 Hz), including the boundary EEG oscillations between δ and θ (3 – 4 Hz), θ and α (7 – 8 Hz), α and β (12 – 13 Hz), β and γ (29 – 30 Hz). For instance, approximately 7.8 Hz in boundary EEG oscillation of θ and α range is one of the candidates for the good cognitive recovery.

c) iEEG protocol for cognitive recovery in neurorehabilitation. The iEEG is comprised of tACS that enhances cognitive training, and monitors of the pre-post EEG changes.

Image 2

A conceptual diagram of a relationship between frequency range of δ-θ, θ-α, α-β, β-γ in EEG and π, π/4 $_{(\times 10)}$, 4π, and $3\pi^2$, respectively.

We speculated that π^2 (≒ 9.86) is the most important number in the perfect round, because 1) 10 Hz (≒ π^2) frequency fulcrum was proposed as the natural frequency of the brain during quiet waking (Garcia-Rill et al., 2016), and 2) it is well known that π^2 (≒ 9.86) is approximately the value (9.8 m/s^2) of the Earth's gravitation acceleration, that seems more than mere coincidence. Therefore, we applied a calculation of isochronous pendulum to create the conceptual diagram of the perfect round using a simple formula of $T = 2\pi\sqrt{L/g}$ (T is a period for one oscillation. L is a length of string of the pendulum and g is the Earth's gravitation acceleration). In history, a seconds

pendulum (a frequency of 0.5 Hz) has a constant length of approximately 1m, meaning that a frequency of a pendulum depends on its length. Therefore, a shorter pendulum will have a higher frequency, such as 1 m ; 0.5 Hz, 1 cm ; 5 Hz, 5mm ; 7.1 Hz, 2.5 mm ; 10 Hz, 1 mm ; 15.8 Hz, respectively. Interestingly, the thickness of the human cerebral cortex is an overall average of approximately 2.5 mm (Fischi et al., 2000), suggesting that a characteristic rhythm of 2.5 mm thickness in the human cerebral cortex corresponds to approximately 10 Hz in the typical alpha-band, and gravity on the Earth might affect the evolution of alpha-rhythm in human brain.

References

Benedek M, Bergner S, Könen T, Fink A, Neubauer AC. (2011). EEG alpha synchronization is related to top-down processing in convergent and divergent thinking. *Neuropsychologia*. 49, 3505-3511. doi: 10.1016/j.neuropsychologia.2011.09.004.

Borwein J.M. (2014) The Life of π: From Archimedes to ENIAC and Beyond. In: Sidoli N., Van Brummelen G. (eds) From Alexandria, Through Baghdad. Springer, Berlin, Heidelberg.

Feurra M, Bianco G, Santarnecchi E, Del Testa M, Rossi A, Rossi S. (2011). Frequency-dependent tuning of the human motor system induced bytranscranial oscillatory potentials. *J Neurosci*. 31, 12165-12170. doi: 10.1523/JNEUROSCI.0978-11.2011.

Fink, A., Benedek, M. (2014). EEG alpha power and creative ideation. *Neurosci Biobehav Rev*. 44, 111-123. doi: 10.1016/j.neubiorev.2012.12.002.

Fischl, B., Dale, A, M. (2000). Measuring the thickness of the human cerebral cortex from magnetic resonance images. *Proc Natl Acad Sci U S A*. 97, 11050-11055.

Garcia-Rill, E., D'Onofrio, S., Luster, B., Mahaffey S, Urbano, F,J., Phillips, C. (2016). The 10 Hz Frequency: A Fulcrum For Transitional Brain States. *Transl Brain Rhythm*.1, 7-13. doi: 10.15761/TBR.1000103

Iaccarino H. F, Singer AC, Martorell A. J, Rudenko A, Gao F, Gillingham T. Z, et al. (2016). Gamma frequency entrainment attenuates amyloid load and modifies microglia. *Nature*. 540, 230-235. doi: 10.1038/nature20587.

Klimesch, W. (1999). EEG alpha and theta oscillations reflect cognitive and memory performance: a review and analysis. Brain Res. Rev. 29, 169–195.

Neubauer, A.C., Wammerl, M., Benedek. M., Jauk, E. and Jausovec. N. (2017). The influence of transcranial alternating current stimulation (tACS) on fluid intelligence: An fMRI study. *Personality and Individual Differences,* 118, 50-55. doi: org/10.1016/j.paid.2017.04.016.

Marshall, L., Helgadóttir, H., Mölle, M., Born J. (2006).Boosting slow oscillations during sleep potentiates memory. *Nature*. 30, 610-613.

Muñoz Garcia, E., Pérez Marco, R. (2008). The Product Over All Primes is $4\pi^2$. *Communications in Mathematical Physics*. 277, 69–81.

Polanía, R., Nitsche, M. A., Korman, C., Batsikadze, G., Paulus, W. (2012). The importance of timing in segregated theta phase-coupling for cognitive performance. *Curr Biol*. **24**,1314-1318. doi: 10.1016/j.cub.2012.05.021.

van Schouwenburg, M.R., Zanto, T.P., Gazzaley, A. (2017). Spatial Attention and the Effects of Frontoparietal Alpha Band Stimulation. *Front Hum Neurosci*. 10:658.

doi: 10.3389/fnhum.2016.00658.

Voss U, Holzmann R, Hobson A, Paulus W, Koppehele-Gossel J, Klimke A, Nitsche MA. (2014). Induction of self awareness in dreams through frontal low current stimulation of gamma activity. *Nat Neurosci*. 17, 810-812 . doi: 10.1038/nn.3719.
