# Supplementary figures and images for: Boundary EEG Asymmetry Is Associated to Linguistic Competence in Vascular Cognitive Impairments
Source: Front Hum Neurosci. 2018 May 9;12:170. doi: 10.3389/fnhum.2018.00170 (PMC5954089; doi:10.3389/fnhum.2018.00170)

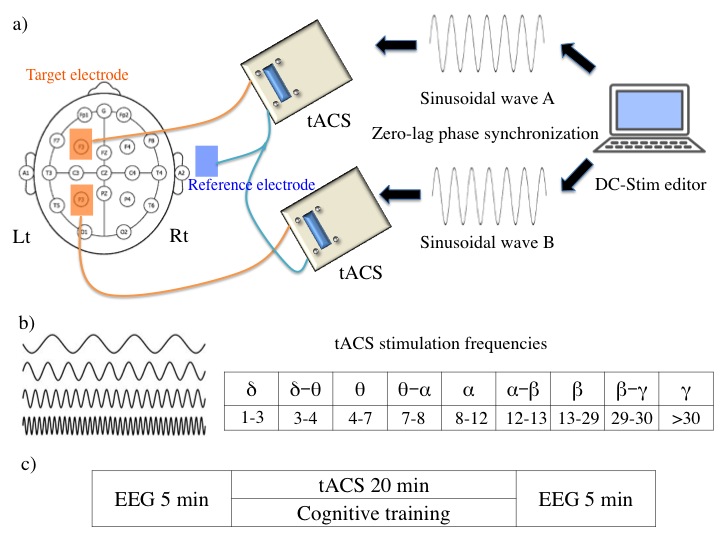

Supplement: Supplementary file 2 [file Image_1.JPEG]

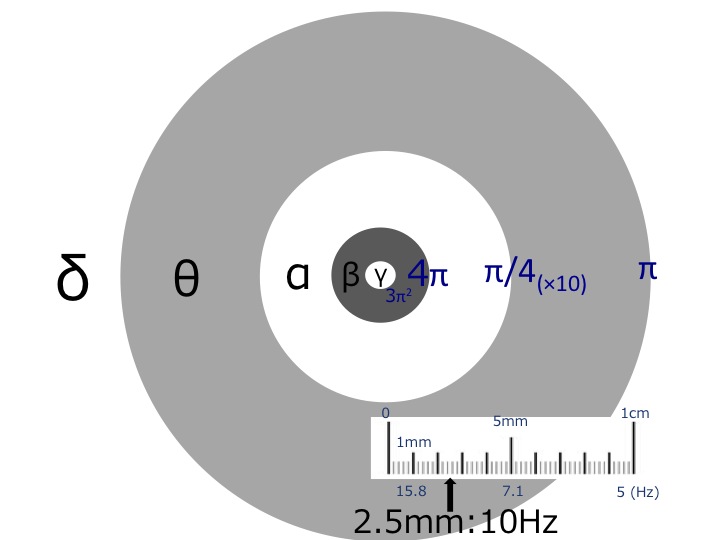

Supplement: Supplementary file 3 [file Image_2.JPEG]
